# 1-Allyl-3,3-di-*p*-tolyl­indolin-2-one

**DOI:** 10.1107/S1600536808010088

**Published:** 2008-04-23

**Authors:** S. Nirmala, E. Theboral Sugi Kamala, L. Sudha, A. R. Naresh Raj, C. A. M. A. Huq

**Affiliations:** a Department of Physics, Easwari Engineering College, Ramapuram, Chennai 600 089, India; b Department of Physics, SRM University, Ramapuram Campus, Chennai 600 089, India; c Post Graduate Research, Department of Chemistry, The New College, Chennai 600 014, India

## Abstract

In the title compound, C_25_H_23_NO, the indoline system is essentially planar. The mol­ecular structure is stabilized by weak intra­molecular C—H⋯N inter­actions and the crystal packing is determined by inter­molecular C—H⋯π inter­actions.

## Related literature

For related literature, see: Harris & Uhle (1960 ▶); Ho *et al.* (1986 ▶); Rajeswaran *et al.* (1999 ▶); Stevenson *et al.* (2000 ▶); Sethusankar *et al.* (2002 ▶).
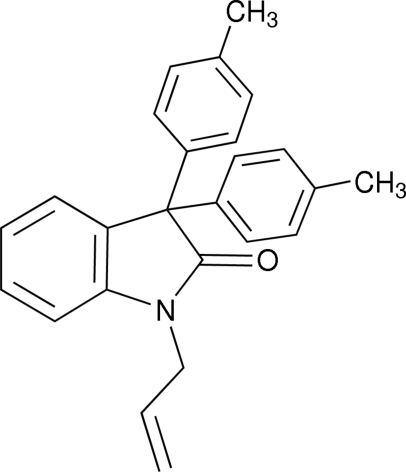


## Experimental

### 

#### Crystal data

C_25_H_23_NO
*M* *_r_* = 353.44
Triclinic, 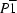

*a* = 9.3311 (2) Å
*b* = 9.5793 (2) Å
*c* = 11.5736 (2) Å
α = 92.163 (1)°
β = 103.192 (1)°
γ = 101.520 (1)°
*V* = 983.15 (3) Å^3^
*Z* = 2
Mo *K*α radiation
μ = 0.07 mm^−1^
*T* = 293 (2) K
0.26 × 0.20 × 0.20 mm

#### Data collection

Bruker Kappa APEXII diffractometer
Absorption correction: multi-scan (Blessing, 1995 ▶) *T*_min_ = 0.982, *T*_max_ = 0.986
25969 measured reflections
6260 independent reflections
4310 reflections with *I* > 2σ(*I*)
*R*_int_ = 0.023

#### Refinement

*R*[*F* ^2^ > 2σ(*F* ^2^)] = 0.051
*wR*(*F* ^2^) = 0.156
*S* = 0.99
6260 reflections
244 parameters
H-atom parameters constrained
Δρ_max_ = 0.24 e Å^−3^
Δρ_min_ = −0.21 e Å^−3^

### 

Data collection: *APEX2* (Bruker, 2004 ▶); cell refinement: *APEX2* and *SAINT* (Bruker, 2004 ▶); data reduction: *SAINT* and *XPREP* (Bruker, 2004 ▶); program(s) used to solve structure: *SHELXS97* (Sheldrick, 2008 ▶); program(s) used to refine structure: *SHELXL97* (Sheldrick, 2008 ▶); molecular graphics: *ORTEP-3* (Farrugia, 1997 ▶); software used to prepare material for publication: *PLATON* (Spek, 2003 ▶).

## Supplementary Material

Crystal structure: contains datablocks I, global. DOI: 10.1107/S1600536808010088/gw2036sup1.cif

Structure factors: contains datablocks I. DOI: 10.1107/S1600536808010088/gw2036Isup2.hkl

Additional supplementary materials:  crystallographic information; 3D view; checkCIF report

## Figures and Tables

**Table 1:** Hydrogen-bond geometry (Å, °)

*D*—H⋯*A*	*D*—H	H⋯*A*	*D*⋯*A*	*D*—H⋯*A*
C5—H5⋯*Cg*^i^	0.93	2.94	3.740 (2)	145
C12—H12*A*⋯N1	0.93	2.54	2.858 (2)	100

Symmetry code: (i) 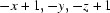. *Cg* denotes the centroid of the C20–C25 ring.

**Fig. 1.**
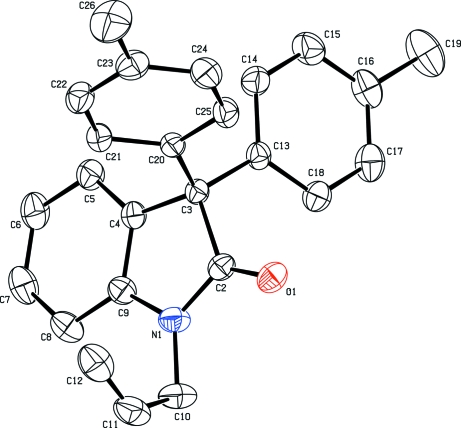
The molecular structure of (I) with 30% probability displacement ellipsoids

**Fig. 2.**
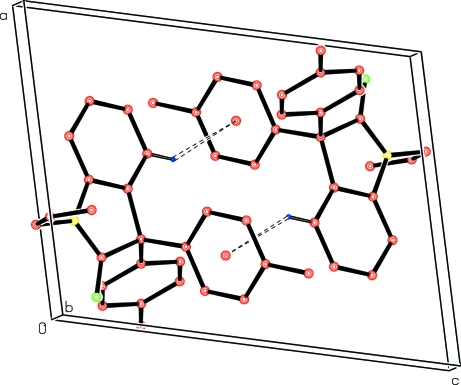
The packing of the molecules viewed down b axis.

## supplementary crystallographic information

### Comment

Indole compounds can be used as bioactive drugs (Stevenson *et al.*, 2000). Indole derivatives exhibit anti-allergic, central nervous system depressant and muscle relaxant properties (Harris & Uhle, 1960; Ho *et al.*, 1986). Indoles have also been proved to display high aldose reductase inhibitory activity (Rajeswaran *et al.*, 1999). In view of this biological importance, an X-ray study of the title compound, (I), was carried out.

An *ORTEP *(Farrugia,1997) plot of the molecular is shown in Fig.1. The indole moiety is planar [maximum deviation of 0.038 (1) from the least square plane defined by all non hydrogen atoms in the molecule] and is nearly orthogonal to methylphenyl rings A and B, and makes a dihedral angle of 72.3 (4)° with the ring A, 76.7 (3)° with the ring B. Both the *p*-tolyl rings A and B are oriented at an angle of 72.6 (4)° with respect to each other. The sum of angles around N1 [360.0]° is in accordance with *sp*^2^ hybridization. The endocyclic angles around C4 is narrowed while those at C9 is widened from 120°. This may be caused by fusion of the smaller pyrrole ring to the six membered benzene ring of oxindole. A similar effect has also been observed by Sethu Sankar *et al.* (2002). The bond lengths in the oxindole ring systems indicate electron delocalization. The torsion angles C19—C16—C15—C14 [–179.5 (2)°], C19—C16—C17—C18 [179.7 (2)°] and C26—C23—C24—C25 [–179.6 (2)°], C26—C23—C22—C21 [179.8 (2)°] indicates that the methyl groups are coplanar with the plane of the attached benzene rings A and B. The allyl group deviates significantly from the plane of the indole moiety [C12—C11—C10—N1 = – 4.5 (3)°].

Weak intramolecular C—H···N and intermolecular C—H··· *Cg* interactions, with C5···*Cg* = 3.740 (2) Å, [*Cg* denotes centroid of C20—C25 ring] are observed in the molecular structure. In addition the packing is stabilized by van der Waals forces.

### Experimental

To a solution of *p*-methyl phenyl magnesium bromide in dry THF, at 0°C under N_2_ atm.,1-*N*-allyl isatin (0.0125 mol, 2.34 g), in dry THF, was added dropwise. After the complete addition, the mixture was stirred at 0°C for 1 hr and then it was stirred at room temperature for 5 hrs. On completion of the reaction, a saturated solution of NH_4_Cl was added slowly at 0°C. The aqueous layer was extracted with ether, and the combined organic layer was extracted with ether. The crude mass was obtained., which was purified over a column of silica gel using hexane/ethyl acetate as eluent. Compound was recrystallized from methanol.

### Refinement

H atoms were positioned geometrically and were treated as riding on their parent C atoms, with aromatic C—H distances of 0.93 Å, methyl C—H distances of 0.96 Å and methylene C—H distances of 0.97 Å, and with *U*_iso_(H) = 1.5*U*_eq_(C) for methyl H and 1.2*U*_eq_(C) for other H atoms.

### Crystal data

**Table:**

C_25_H_23_NO	*Z* = 2
*M**_r_* = 353.44	*F*_000_ = 376
Triclinic, *P*1	*D*_x_ = 1.194 Mg m^−^^3^
Hall symbol: -P 1	Mo *K*α radiation λ = 0.71073 Å
*a* = 9.3311 (2) Å	Cell parameters from 9112 reflections
*b* = 9.5793 (2) Å	θ = 2.3–30.1º
*c* = 11.5736 (2) Å	µ = 0.07 mm^−^^1^
α = 92.1630 (10)º	*T* = 293 (2) K
β = 103.1920 (10)º	Prism, colourless
γ = 101.5200 (10)º	0.26 × 0.20 × 0.20 mm
*V* = 983.15 (3) Å^3^	

### Data collection

**Table:**

Bruker Kappa APEXII diffractometer	6260 independent reflections
Radiation source: fine-focus sealed tube	4310 reflections with *I* > 2σ(*I*)
Monochromator: graphite	*R*_int_ = 0.023
*T* = 293(2) K	θ_max_ = 31.0º
ω and φ scan	θ_min_ = 2.2º
Absorption correction: multi-scan(Blessing, 1995)	*h* = −13→13
*T*_min_ = 0.982, *T*_max_ = 0.986	*k* = −13→13
25969 measured reflections	*l* = −16→16

### Refinement

**Table:**

Refinement on *F*^2^	Secondary atom site location: difference Fourier map
Least-squares matrix: full	Hydrogen site location: inferred from neighbouring sites
*R*[*F*^2^ > 2σ(*F*^2^)] = 0.051	H-atom parameters constrained
*wR*(*F*^2^) = 0.156	*w* = 1/[σ^2^(*F*_o_^2^) + (0.0731*P*)^2^ + 0.1889*P*] where *P* = (*F*_o_^2^ + 2*F*_c_^2^)/3
*S* = 1.00	(Δ/σ)_max_ < 0.001
6260 reflections	Δρ_max_ = 0.25 e Å^−^^3^
244 parameters	Δρ_min_ = −0.21 e Å^−^^3^
Primary atom site location: structure-invariant direct methods	Extinction correction: none

### Special details

**Table:**

Geometry. All e.s.d.'s (except the e.s.d. in the dihedral angle between two l.s. planes) are estimated using the full covariance matrix. The cell e.s.d.'s are taken into account individually in the estimation of e.s.d.'s in distances, angles and torsion angles; correlations between e.s.d.'s in cell parameters are only used when they are defined by crystal symmetry. An approximate (isotropic) treatment of cell e.s.d.'s is used for estimating e.s.d.'s involving l.s. planes.
Refinement. Refinement of *F*^2^ against ALL reflections. The weighted *R*-factor *wR* and goodness of fit *S* are based on *F*^2^, conventional *R*-factors *R* are based on *F*, with *F* set to zero for negative *F*^2^. The threshold expression of *F*^2^ > σ(*F*^2^) is used only for calculating *R*-factors(gt) *etc*. and is not relevant to the choice of reflections for refinement. *R*-factors based on *F*^2^ are statistically about twice as large as those based on *F*, and *R*- factors based on ALL data will be even larger.

### Fractional atomic coordinates and isotropic or equivalent isotropic displacement parameters (Å^2^)

**Table:**

	*x*	*y*	*z*	*U*_iso_*/*U*_eq_	
C2	0.21981 (13)	0.10769 (13)	0.14416 (10)	0.0403 (2)	
C3	0.29661 (11)	0.03653 (12)	0.25302 (9)	0.0361 (2)	
C4	0.45434 (12)	0.05217 (12)	0.23447 (10)	0.0391 (2)	
C5	0.57669 (13)	0.00390 (15)	0.29710 (12)	0.0497 (3)	
H5	0.5699	−0.0478	0.3628	0.060*	
C6	0.71037 (15)	0.03411 (17)	0.26019 (15)	0.0602 (4)	
H6	0.7938	0.0024	0.3018	0.072*	
C7	0.72086 (16)	0.10999 (18)	0.16332 (16)	0.0629 (4)	
H7	0.8116	0.1295	0.1405	0.075*	
C8	0.59809 (17)	0.15830 (17)	0.09859 (14)	0.0587 (4)	
H8	0.6049	0.2096	0.0327	0.070*	
C9	0.46580 (14)	0.12724 (13)	0.13582 (11)	0.0443 (3)	
C10	0.29599 (19)	0.24323 (18)	−0.01709 (12)	0.0614 (4)	
H10A	0.1917	0.2102	−0.0604	0.074*	
H10B	0.3589	0.2267	−0.0701	0.074*	
C11	0.32409 (19)	0.39967 (19)	0.01642 (16)	0.0658 (4)	
H11	0.3133	0.4583	−0.0458	0.079*	
C12	0.3620 (2)	0.4623 (2)	0.12318 (19)	0.0749 (5)	
H12A	0.3742	0.4084	0.1885	0.090*	
H12B	0.3770	0.5612	0.1347	0.090*	
C13	0.22090 (12)	−0.12027 (12)	0.25034 (10)	0.0391 (2)	
C14	0.24490 (16)	−0.19198 (15)	0.35239 (13)	0.0541 (3)	
H14	0.3029	−0.1423	0.4237	0.065*	
C15	0.18392 (18)	−0.33608 (16)	0.34970 (16)	0.0626 (4)	
H15	0.2020	−0.3818	0.4194	0.075*	
C16	0.09700 (17)	−0.41371 (15)	0.24620 (16)	0.0592 (4)	
C17	0.07309 (17)	−0.34194 (16)	0.14492 (15)	0.0603 (4)	
H17	0.0145	−0.3920	0.0739	0.072*	
C18	0.13362 (15)	−0.19778 (14)	0.14583 (12)	0.0490 (3)	
H18	0.1156	−0.1526	0.0759	0.059*	
C19	0.0294 (3)	−0.57073 (17)	0.2435 (2)	0.0907 (6)	
H19A	−0.0262	−0.6064	0.1639	0.136*	
H19B	−0.0372	−0.5842	0.2963	0.136*	
H19C	0.1083	−0.6216	0.2686	0.136*	
C20	0.28928 (12)	0.12652 (12)	0.36267 (10)	0.0372 (2)	
C21	0.40937 (13)	0.23204 (14)	0.42458 (11)	0.0445 (3)	
H21	0.5003	0.2467	0.4020	0.053*	
C22	0.39541 (16)	0.31600 (15)	0.51984 (12)	0.0530 (3)	
H22	0.4778	0.3858	0.5605	0.064*	
C23	0.26275 (17)	0.29871 (17)	0.55575 (12)	0.0553 (3)	
C24	0.14184 (16)	0.19372 (17)	0.49283 (13)	0.0561 (3)	
H24	0.0506	0.1801	0.5150	0.067*	
C25	0.15471 (14)	0.10923 (15)	0.39803 (12)	0.0483 (3)	
H25	0.0721	0.0398	0.3573	0.058*	
C26	0.2485 (3)	0.3901 (3)	0.65992 (18)	0.0932 (7)	
H26A	0.1485	0.3626	0.6718	0.140*	
H26B	0.2675	0.4889	0.6439	0.140*	
H26C	0.3204	0.3771	0.7304	0.140*	
N1	0.32609 (13)	0.15965 (12)	0.08421 (9)	0.0484 (3)	
O1	0.08971 (10)	0.11949 (10)	0.11825 (8)	0.0515 (2)	

### Atomic displacement parameters (Å^2^)

**Table:**

	*U*^11^	*U*^22^	*U*^33^	*U*^12^	*U*^13^	*U*^23^
C2	0.0417 (5)	0.0399 (6)	0.0385 (5)	0.0124 (4)	0.0053 (4)	0.0024 (4)
C3	0.0306 (5)	0.0386 (6)	0.0381 (5)	0.0075 (4)	0.0059 (4)	0.0061 (4)
C4	0.0342 (5)	0.0399 (6)	0.0429 (6)	0.0077 (4)	0.0094 (4)	0.0027 (4)
C5	0.0372 (6)	0.0554 (8)	0.0569 (7)	0.0132 (5)	0.0086 (5)	0.0103 (6)
C6	0.0364 (6)	0.0650 (9)	0.0805 (10)	0.0145 (6)	0.0135 (6)	0.0057 (8)
C7	0.0444 (7)	0.0637 (9)	0.0859 (11)	0.0079 (6)	0.0305 (7)	0.0025 (8)
C8	0.0586 (8)	0.0602 (9)	0.0656 (9)	0.0114 (7)	0.0317 (7)	0.0128 (7)
C9	0.0444 (6)	0.0442 (6)	0.0468 (6)	0.0109 (5)	0.0148 (5)	0.0051 (5)
C10	0.0757 (10)	0.0732 (10)	0.0455 (7)	0.0298 (8)	0.0202 (7)	0.0229 (7)
C11	0.0697 (9)	0.0666 (10)	0.0719 (10)	0.0222 (8)	0.0280 (8)	0.0323 (8)
C12	0.0729 (11)	0.0612 (10)	0.0954 (13)	0.0132 (8)	0.0299 (10)	0.0153 (9)
C13	0.0318 (5)	0.0389 (6)	0.0461 (6)	0.0085 (4)	0.0074 (4)	0.0048 (5)
C14	0.0522 (7)	0.0470 (7)	0.0556 (7)	0.0060 (6)	0.0005 (6)	0.0122 (6)
C15	0.0626 (9)	0.0476 (8)	0.0785 (10)	0.0132 (6)	0.0152 (7)	0.0224 (7)
C16	0.0542 (7)	0.0368 (7)	0.0920 (11)	0.0108 (6)	0.0280 (7)	0.0023 (7)
C17	0.0582 (8)	0.0462 (8)	0.0716 (9)	0.0058 (6)	0.0133 (7)	−0.0137 (7)
C18	0.0499 (7)	0.0470 (7)	0.0480 (6)	0.0096 (5)	0.0092 (5)	−0.0022 (5)
C19	0.0989 (14)	0.0380 (8)	0.1420 (19)	0.0073 (8)	0.0500 (14)	0.0009 (10)
C20	0.0339 (5)	0.0404 (6)	0.0373 (5)	0.0077 (4)	0.0078 (4)	0.0079 (4)
C21	0.0372 (5)	0.0475 (7)	0.0461 (6)	0.0038 (5)	0.0097 (5)	0.0032 (5)
C22	0.0521 (7)	0.0519 (8)	0.0491 (7)	0.0055 (6)	0.0063 (6)	−0.0046 (6)
C23	0.0607 (8)	0.0625 (9)	0.0456 (7)	0.0186 (7)	0.0145 (6)	0.0007 (6)
C24	0.0486 (7)	0.0701 (9)	0.0558 (7)	0.0148 (6)	0.0229 (6)	0.0056 (7)
C25	0.0361 (5)	0.0550 (7)	0.0522 (7)	0.0046 (5)	0.0121 (5)	0.0027 (6)
C26	0.0965 (14)	0.1150 (17)	0.0707 (11)	0.0262 (12)	0.0279 (10)	−0.0268 (11)
N1	0.0520 (6)	0.0551 (6)	0.0434 (5)	0.0188 (5)	0.0140 (5)	0.0154 (5)
O1	0.0433 (4)	0.0595 (6)	0.0515 (5)	0.0207 (4)	0.0023 (4)	0.0074 (4)

### Geometric parameters (Å, °)

**Table:**

C2—O1	1.2114 (14)	C14—C15	1.380 (2)
C2—N1	1.3621 (16)	C14—H14	0.9300
C2—C3	1.5535 (15)	C15—C16	1.376 (2)
C3—C4	1.5143 (15)	C15—H15	0.9300
C3—C13	1.5228 (16)	C16—C17	1.378 (2)
C3—C20	1.5294 (16)	C16—C19	1.507 (2)
C4—C5	1.3788 (16)	C17—C18	1.382 (2)
C4—C9	1.3853 (17)	C17—H17	0.9300
C5—C6	1.3895 (19)	C18—H18	0.9300
C5—H5	0.9300	C19—H19A	0.9600
C6—C7	1.371 (2)	C19—H19B	0.9600
C6—H6	0.9300	C19—H19C	0.9600
C7—C8	1.391 (2)	C20—C21	1.3847 (16)
C7—H7	0.9300	C20—C25	1.3885 (16)
C8—C9	1.3798 (19)	C21—C22	1.3857 (19)
C8—H8	0.9300	C21—H21	0.9300
C9—N1	1.4046 (16)	C22—C23	1.375 (2)
C10—N1	1.4508 (16)	C22—H22	0.9300
C10—C11	1.489 (2)	C23—C24	1.390 (2)
C10—H10A	0.9700	C23—C26	1.509 (2)
C10—H10B	0.9700	C24—C25	1.380 (2)
C11—C12	1.293 (3)	C24—H24	0.9300
C11—H11	0.9300	C25—H25	0.9300
C12—H12A	0.9300	C26—H26A	0.9600
C12—H12B	0.9300	C26—H26B	0.9600
C13—C18	1.3852 (17)	C26—H26C	0.9600
C13—C14	1.3853 (17)		
			
O1—C2—N1	125.45 (11)	C16—C15—C14	121.51 (14)
O1—C2—C3	126.57 (11)	C16—C15—H15	119.2
N1—C2—C3	107.95 (9)	C14—C15—H15	119.2
C4—C3—C13	111.14 (9)	C15—C16—C17	117.38 (13)
C4—C3—C20	113.55 (9)	C15—C16—C19	121.49 (17)
C13—C3—C20	112.94 (9)	C17—C16—C19	121.13 (17)
C4—C3—C2	101.28 (9)	C16—C17—C18	121.93 (14)
C13—C3—C2	111.74 (9)	C16—C17—H17	119.0
C20—C3—C2	105.46 (9)	C18—C17—H17	119.0
C5—C4—C9	119.89 (11)	C17—C18—C13	120.39 (13)
C5—C4—C3	130.70 (11)	C17—C18—H18	119.8
C9—C4—C3	109.40 (9)	C13—C18—H18	119.8
C4—C5—C6	118.68 (13)	C16—C19—H19A	109.5
C4—C5—H5	120.7	C16—C19—H19B	109.5
C6—C5—H5	120.7	H19A—C19—H19B	109.5
C7—C6—C5	120.88 (13)	C16—C19—H19C	109.5
C7—C6—H6	119.6	H19A—C19—H19C	109.5
C5—C6—H6	119.6	H19B—C19—H19C	109.5
C6—C7—C8	121.13 (13)	C21—C20—C25	117.95 (11)
C6—C7—H7	119.4	C21—C20—C3	122.56 (10)
C8—C7—H7	119.4	C25—C20—C3	119.36 (10)
C9—C8—C7	117.48 (14)	C20—C21—C22	120.67 (12)
C9—C8—H8	121.3	C20—C21—H21	119.7
C7—C8—H8	121.3	C22—C21—H21	119.7
C8—C9—C4	121.93 (12)	C23—C22—C21	121.62 (13)
C8—C9—N1	128.51 (12)	C23—C22—H22	119.2
C4—C9—N1	109.54 (10)	C21—C22—H22	119.2
N1—C10—C11	113.55 (13)	C22—C23—C24	117.66 (13)
N1—C10—H10A	108.9	C22—C23—C26	121.35 (15)
C11—C10—H10A	108.9	C24—C23—C26	120.99 (15)
N1—C10—H10B	108.9	C25—C24—C23	121.17 (13)
C11—C10—H10B	108.9	C25—C24—H24	119.4
H10A—C10—H10B	107.7	C23—C24—H24	119.4
C12—C11—C10	126.66 (15)	C24—C25—C20	120.91 (12)
C12—C11—H11	116.7	C24—C25—H25	119.5
C10—C11—H11	116.7	C20—C25—H25	119.5
C11—C12—H12A	120.0	C23—C26—H26A	109.5
C11—C12—H12B	120.0	C23—C26—H26B	109.5
H12A—C12—H12B	120.0	H26A—C26—H26B	109.5
C18—C13—C14	117.85 (12)	C23—C26—H26C	109.5
C18—C13—C3	121.72 (11)	H26A—C26—H26C	109.5
C14—C13—C3	120.33 (10)	H26B—C26—H26C	109.5
C15—C14—C13	120.95 (13)	C2—N1—C9	111.71 (10)
C15—C14—H14	119.5	C2—N1—C10	122.67 (11)
C13—C14—H14	119.5	C9—N1—C10	125.57 (12)
			
O1—C2—C3—C4	178.68 (12)	C14—C15—C16—C17	0.0 (2)
N1—C2—C3—C4	−3.41 (12)	C14—C15—C16—C19	−179.48 (16)
O1—C2—C3—C13	60.29 (16)	C15—C16—C17—C18	0.2 (2)
N1—C2—C3—C13	−121.80 (11)	C19—C16—C17—C18	179.68 (15)
O1—C2—C3—C20	−62.78 (15)	C16—C17—C18—C13	−0.2 (2)
N1—C2—C3—C20	115.14 (10)	C14—C13—C18—C17	0.1 (2)
C13—C3—C4—C5	−57.30 (16)	C3—C13—C18—C17	176.45 (12)
C20—C3—C4—C5	71.33 (16)	C4—C3—C20—C21	11.87 (15)
C2—C3—C4—C5	−176.12 (13)	C13—C3—C20—C21	139.58 (11)
C13—C3—C4—C9	121.92 (11)	C2—C3—C20—C21	−98.13 (12)
C20—C3—C4—C9	−109.45 (11)	C4—C3—C20—C25	−172.36 (10)
C2—C3—C4—C9	3.10 (12)	C13—C3—C20—C25	−44.66 (14)
C9—C4—C5—C6	1.0 (2)	C2—C3—C20—C25	77.64 (13)
C3—C4—C5—C6	−179.88 (12)	C25—C20—C21—C22	0.88 (18)
C4—C5—C6—C7	−0.2 (2)	C3—C20—C21—C22	176.71 (11)
C5—C6—C7—C8	−0.4 (2)	C20—C21—C22—C23	−0.5 (2)
C6—C7—C8—C9	0.2 (2)	C21—C22—C23—C24	−0.2 (2)
C7—C8—C9—C4	0.7 (2)	C21—C22—C23—C26	179.75 (16)
C7—C8—C9—N1	−177.86 (14)	C22—C23—C24—C25	0.4 (2)
C5—C4—C9—C8	−1.2 (2)	C26—C23—C24—C25	−179.55 (16)
C3—C4—C9—C8	179.45 (12)	C23—C24—C25—C20	0.1 (2)
C5—C4—C9—N1	177.52 (12)	C21—C20—C25—C24	−0.69 (19)
C3—C4—C9—N1	−1.79 (14)	C3—C20—C25—C24	−176.65 (12)
N1—C10—C11—C12	−4.5 (3)	O1—C2—N1—C9	−179.43 (12)
C4—C3—C13—C18	−91.47 (13)	C3—C2—N1—C9	2.63 (14)
C20—C3—C13—C18	139.57 (11)	O1—C2—N1—C10	3.1 (2)
C2—C3—C13—C18	20.86 (15)	C3—C2—N1—C10	−174.88 (12)
C4—C3—C13—C14	84.82 (14)	C8—C9—N1—C2	178.07 (13)
C20—C3—C13—C14	−44.14 (14)	C4—C9—N1—C2	−0.58 (15)
C2—C3—C13—C14	−162.84 (11)	C8—C9—N1—C10	−4.5 (2)
C18—C13—C14—C15	0.1 (2)	C4—C9—N1—C10	176.83 (13)
C3—C13—C14—C15	−176.30 (13)	C11—C10—N1—C2	88.62 (17)
C13—C14—C15—C16	−0.2 (2)	C11—C10—N1—C9	−88.53 (17)

### Hydrogen-bond geometry (Å, °)

**Table:**

*D*—H···*A*	*D*—H	H···*A*	*D*···*A*	*D*—H···*A*
C5—H5···Cg^i^	0.93	2.94	3.740 (2)	145
C12—H12A···N1	0.93	2.54	2.858 (2)	100

Symmetry codes: (i) −*x*+1, −*y*, −*z*+1.

## References

[bb1] Blessing, R. H. (1995). *Acta Cryst.* A**51**, 33–38. 10.1107/s0108767394005726 7702794

[bb2] Bruker (2004). *APEX2*, *SAINT* and *XPREP* Bruker AXS Inc., Madison, Wisconsin, USA.

[bb3] Farrugia, L. J. (1997). *J. Appl. Cryst.* **30**, 565.

[bb4] Harris, L. S. & Uhle, F. C. (1960). *J. Pharmacol. Exp. Ther.* **128**, 353–363. 14399979

[bb5] Ho, C. Y., Haegman, W. E. & Perisco, F. (1986). *J. Med. Chem.* **29**, 118–121.

[bb6] Rajeswaran, W. G., Labroo, R. B., Cohen, L. A. & King, M. M. (1999). *J. Org. Chem.* **64**, 1369–1371.

[bb7] Sethu Sankar, K., Kannadasan, S., Velmurugan, D., Srinivasan, P. C., Shanmuga Sundara Raj, S. & Fun, H.-K. (2002). *Acta Cryst.* C**58**, o277–o279. 10.1107/s0108270102005152 11983990

[bb8] Sheldrick, G. M. (2008). *Acta Cryst.* A**64**, 112–122. 10.1107/S0108767307043930 18156677

[bb9] Spek, A. L. (2003). *J. Appl. Cryst.* **36**, 7–13.

[bb10] Stevenson, G. I., Smith, A. L., Lewis, S., Michie, S. G., Neduvelil, J. G., Patel, S., Marwood, R., Patel, S. & Castro, J. L. (2000). *Bioorg. Med. Chem. Lett.* **10**, 2697–2704. 10.1016/s0960-894x(00)00557-6 11133071

